# Increased risk of incident diabetes in patients with MAFLD not meeting the criteria for NAFLD

**DOI:** 10.1038/s41598-023-37858-8

**Published:** 2023-07-01

**Authors:** So Hee Park, Jiyun Park, So Yoon Kwon, You-Bin Lee, Gyuri Kim, Kyu Yeon Hur, Janghyun Koh, Jae Hwan Jee, Jae Hyeon Kim, Mira Kang, Sang-Man Jin

**Affiliations:** 1 Division of Endocrinology and Metabolism, Department of Medicine, Samsung Medical Center, Sungkyunkwan University School of Medicine, 81 Irwon-ro, Gangnam-gu, Seoul, 06351 Republic of Korea; 2 Division of Endocrine and Metabolism, Department of Internal Medicine, CHA Bundang Medical Center, CHA University School of Medicine, 59 Yatap-ro, Bundang-gu, Seongnam, Gyeonggi-do 14396 Republic of Korea; 3 Department of Health Promotion Center, Center for Health Promotion, Samsung Medical Center, Sungkyunkwan University School of Medicine, 81, Irwon-ro, Gangnam-gu, Seoul, 06351 Republic of Korea; 4 Department of Digital Health, SAIHST, Sungkyunkwan University, Seoul, Republic of Korea

**Keywords:** Diabetes, Non-alcoholic fatty liver disease

## Abstract

We aimed to compare the risk of incident diabetes according to fatty liver disease (FLD) definition, focusing on the comparison between those who met criteria for either metabolic dysfunction-associated fatty liver disease (MAFLD) or nonalcoholic fatty liver disease (NAFLD) but not the other. This was a 5.0-year (interquartile range, 2.4–8.2) retrospective longitudinal cohort study of 21,178 adults who underwent at least two serial health checkup examinations. The presence of hepatic steatosis was determined by abdominal ultrasonography at the first health examination. Cox proportional hazard analyses were used to compare the risk of incident diabetes among five groups. Incident diabetes cases occurred in 1296 participants (6.1%). When non-FLD without metabolic dysfunction (MD) group was set as a reference, the risk of incident diabetes increased in the order of NAFLD-only, non-FLD with MD, both FLD, and MAFLD-only groups. The presence of excessive alcohol consumption and/or hepatitis B virus (HBV)/hepatitis C virus (HCV) infection, FLD, and MD synergistically increased the risk of incident diabetes. MAFLD-only group showed a greater increase in incidence of diabetes than non-FLD with MD and NAFLD-only groups. The interaction among excessive alcohol consumption, HBV/HCV infection, MD, and hepatic steatosis on the development of diabetes should not be overlooked.

## Introduction

Nonalcoholic fatty liver disease (NAFLD) is defined as a spectrum of progressive liver diseases, including steatosis, steatohepatitis, fibrosis, and cirrhosis, that are not due to heavy alcohol consumption or other known causes of liver disease^1,2^. NAFLD is associated with increased risk of diabetes^3–6^ in a complex and bidirectional manner^7,8^. Interactions among hepatic inflammation and fibrosis, atherogenic dyslipidemia, insulin resistance induced by hepatic fat accumulation^9,10^, and systemic release of pro-inflammatory cytokines^11^ and hepatokines such as fetuin A, fetuin B, selenoprotein, and fibroblast growth factor 21^12,13^ are involved in the bidirectional association between diabetes and NAFLD^14^.

Metabolic dysfunction–associated fatty liver disease (MAFLD) is defined as evidence of hepatic steatosis with at least one of the following conditions: overweight/obesity, diabetes or metabolic dysfunction (MD)^15^. While the definition of NAFLD excludes fatty liver disease with heavy alcohol consumption or hepatitis B virus (HBV)/hepatitis C virus (HCV) infection^16^, the definition of MAFLD includes patients with hepatic steatosis combined with excessive alcohol consumption or HBV/HCV infection^17^. This is an important distinction in terms of the pathophysiology of fatty liver disease (FLD), allowing consideration of multiple etiologies that can exist simultaneously^18^. In fact, when the concept of MAFLD was initially introduced, excessive alcohol consumption and HBV/HCV infection were included to capture the notion of multiple etiologies. However, since those with HBV/HCV infection or excessive alcohol intake have been excluded from studies evaluating NAFLD, few studies have addressed the association between HBV/HCV infection- or excessive alcohol consumption-associated FLD and extrahepatic outcomes such as cardiovascular diseases and diabetes^19–23^.

Even without FLD, heavy alcohol consumption^23–25^, HBV^26,27^ and HCV infection^28^ have been suggested as risk factors for diabetes. In this context, it would be meaningful to evaluate the risk of diabetes in those who met the criteria for MAFLD but not NAFLD, because most of them would have a second etiology for FLD such as HBV/HCV infection or excessive alcohol consumption. Although there are few studies on the association between MAFLD and incident diabetes^17,29^, most of them explored the association between MAFLD as a conglomerate and the risk of incident diabetes, rather than the association between each component of MAFLD and the risk of incident diabetes. Unlike in NAFLD, the relative contributions of hepatic steatosis *per se* and of each component of metabolic dysfunction included in the MAFLD definition to the risk of incident diabetes has not been determined in those with HBV/HCV infection- or excessive alcohol consumption-associated FLD.

Therefore, this study was conducted to compare the risk of incident diabetes according to FLD definition, focusing on the comparison between those who met criteria for either MAFLD or NAFLD but not the other, using a large-scale community-based dataset in which presence of hepatic steatosis was determined by abdominal ultrasonography. In addition, the incidence of diabetes was investigated according to excessive alcohol consumption, HBV/HCV infection, MD, and hepatic steatosis to identify a synergistic effect of those components on development of diabetes.

## Results

### Baseline characteristics of the study population

Among the 21,178 individuals in the total study group, 10,505 (49.60%) did not have fatty liver and MD (non-FLD without MD group) and 3768 (17.79%) did not have fatty liver and had MD (non-FLD with MD group). The remaining 6905 individuals had fatty liver, of which 718 (3.39%) met only the MAFLD criteria (MAFLD-only group), 512 (2.42%) met only the NAFLD criteria (NAFLD-only group), 5640 (26.63%) met both the MAFLD and NAFLD criteria (both FLD group), and 35 (0.17%) met neither the MAFLD nor NAFLD criteria. Baseline characteristics of the five groups are presented in Table 1.

**Table 1:** Baseline characteristics of patients included in the study.

Characteristics	Non-FLD without MD	Non-FLD with MD	MAFLD-only	NAFLD-only	Both FLD	*p *value
*N* (%)	10,505 (49.60)	3768 (17.79)	718 (3.39)	512 (2.42)	5640 (26.63)	
Age, yrs	43 ± 9.22	48 ± 8.89	47 ± 8.10	45 ± 8.97	47 ± 8.59	< 0.01
Male sex, *n* (%)	5713 (54.38)	2852 (75.69)	679 (94.57)	387 (75.59)	5063 (89.77)	< 0.01
BMI, kg/m^2^	22.04 ± 2.37	24.41 ± 2.45	26.42 ± 2.71	21.71 ± 1.09	25.95 ± 2.44	< 0.001
Excess drinker, *n* (%)^†^	380 (3.62)	277 (7.35)	429 (59.75)	0 (0)	0 (0)	< 0.01
Alcohol amount, g/day	8.21 ± 12.22	13.09 ± 15.68	39.38 ± 28.71	6.76 ± 7.75	9.97 ± 9.01	< 0.01
Alcohol duration, yrs	19.66 ± 9.47	24.50 ± 9.59	25.52 ± 8.80	23.26 ± 9.15	24.27 ± 8.51	< 0.01
Hepatitis B, *n* (%)^†^	512 (4.87)	208 (5.52)	275 (38.30)	0 (0)	0 (0)	< 0.01
Hepatitis C, *n* (%)^†^	58 (0.55)	24 (0.64)	31 (4.32)	0 (0)	0 (0)	< 0.01
Use of antiviral agents, *n* (%)	113 (1.08)	54 (1.43)	76 (10.58)	0 (0)	0 (0)	< 0.01
Current smoker, *n* (%)	2286 (21.76)	1112 (29.51)	310 (43.18)	142 (27.73)	1912 (33.90)	< 0.01
Comorbidities, *n* (%)						< 0.01
Prediabetes^†^	975 (9.28)	1873 (49.71)	337 (46.94)	75 (14.65)	2321 (41.15)	
Hypertension^†^	1407 (13.39)	2376 (63.06)	369 (51.39)	77 (15.04)	2752 (48.79)	
CKD^†^	107 (1.02)	136 (3.61)	18 (2.51)	5 (0.98)	154 (2.73)	
WC, cm						
Men	82.61 ± 5.96	88.65 ± 6.42	92.12 ± 6.70	81.78 ± 4.25	91.06 ± 6.42	< 0.01
Women	72.91 ± 6.05	80.30 ± 6.51	87.63 ± 6.96	73.67 ± 5.26	83.81 ± 7.56	< 0.01
BUN, mg/dl	12.83 ± 3.28	13.46 ± 3.51	13.71 ± 3.15	13.13 ± 3.06	13.79 ± 3.26	< 0.01
Creatinine, mg/dl	0.88 ± 0.18	0.95 ± 0.19	0.97 ± 0.14	0.92 ± 0.16	0.99 ± 0.17	< 0.01
eGFR, ml/min/1.73m^2^	95.29 ± 13.94	90.04 ± 14.22	91.16 ± 12.95	93.73 ± 13.21	89.68 ± 13.79	< 0.01
uACR, μg/mgCr	7.94 ± 43.62	15.02 ± 71.96	11.04 ± 36.58	7.27 ± 17.59	12.88 ± 76.29	< 0.01
AST, IU/L	20.95 ± 11.89	22.95 ± 13.44	30.55 ± 25.99	22.66 ± 18.53	26.02 ± 13.28	< 0.01
ALT, IU/L	19.43 ± 17.04	24.38 ± 16.52	38.38 ± 34.92	24.89 ± 15.14	34.87 ± 24.24	< 0.01
FPG, mg/dl	87.41 ± 7.60	94.63 ± 9.55	96.14 ± 10.24	89.49 ± 7.78	94.43 ± 9.65	< 0.01
HbA1c, %	5.16 ± 0.36	5.38 ± 0.42	5.39 ± 0.41	5.23 ± 0.37	5.38 ± 0.42	< 0.01
HOMA-IR	1.34 ± 0.68	2.01 ± 1.03	2.47 ± 1.42	1.49 ± 0.72	2.38 ± 1.29	< 0.01
TGs, mg/dl	85.36 ± 37.10	141.17 ± 78.66	167.65 ± 116.35	107.36 ± 49.55	162.63 ± 89.36	< 0.01
HDL-C, mg/dl	62.15 ± 14.45	52.63 ± 13.64	51.37 ± 13.08	56.85 ± 12.87	48.82 ± 10.64	< 0.01
hs-CRP, mg/dl	0.09 ± 0.22	0.16 ± 0.55	0.18 ± 0.45	0.10 ± 0.16	0.17 ± 0.54	< 0.01
NFS	− 2.83 ± 0.98	− 2.30 ± 1.02	− 2.26 ± 1.02	− 3.02 ± 1.01	− 2.51 ± 1.04	< 0.01
US steatosis severity, *n* (%)						
Normal	10,505 (100)	3768 (100)	0 (0)	0 (0)	0 (0)	
Mild	0 (0)	0 (0)	446 (62.12)	414 (80.86)	3390 (60.11)	
Moderate	0 (0)	0 (0)	251 (34.96)	94 (18.36)	2055 (36.44)	
Severe	0 (0)	0 (0)	21 (2.92)	4 (0.78)	195 (3.46)	< 0.01

Data are shown as mean ± standard deviations or frequency as appropriate. *P*-value was calculated using one-way ANOVA test for continuous variables and Chi-square test for categorical variables. The Scheffe test was used in post-hoc analysis.

Abbreviations: *BMI* Body mass index; *CKD* Chronic kidney disease; *WC* Waist circumference; *eGFR* Estimated glomerular filtration rate; *uACR* Urinary albumin-to-creatinine ratio; *AST* aspartate aminotransferase; *ALT* Alanine aminotransferase; *FPG* Fasting plasma glucose; *HbA1c* Glycated hemoglobin; *HOMA-IR* Homeostasis model assessment of insulin resistance; *TGs* Triglycerides; *HDL-C* High-density lipoprotein-cholesterol; *hs-CRP* High-sensitivity C-reactive protein; *FLD* Fatty liver disease; *MD* Metabolic dysfunction; *MAFLD* Metabolic dysfunction–associated fatty liver disease; *NAFLD* Nonalcoholic fatty liver disease; *NFS* NAFLD fibrosis score, *US* Ultrasonography; *HBsAg* Hepatitis B virus surface antigen; *anti-HCV Ab* Hepatitis C antibody.

^†^Overweight/obesity: BMI of 23.0 or greater; hypertension: blood pressure greater than or equal to 130/85 mm Hg or specific drug treatment; prediabetes: fasting glucose 100 to 125 mg/dl or HbA1c 5.7% to 6.4% in participants without a prior diabetes diagnosis; CKD: eGFR less than 60 mL/min/1.73 m^2^ and/or uACR greater than or equal to 30 mg/g; Excess drinker was defined as more than 30 g daily of alcohol consumption in men and more than 20 g in women; Hepatitis B was defined as positive HBsAg or a history of antiviral treatment; Hepatitis C was defined as positive anti-HCV antibody or a history of antiviral treatment.

The non-FLD with MD, MAFLD-only, and both FLD groups tend to have a higher prevalence of males, obesity, prediabetes, hypertension and chronic kidney disease (CKD) compared with the NAFLD-only and non-FLD without MD groups. In addition, participants in the non-FLD with MD, MAFLD-only, and both FLD groups had a lower estimated glomerular filtration rate (eGFR) and higher urinary albumin-to-creatinine ratio (uACR), fasting plasma glucose (FPG), glycosylated hemoglobin (HbA1c), homeostasis model assessment of insulin resistance (HOMA-IR), triglycerides (TGs), high-sensitivity C-reactive protein (hs-CRP) and nonalcoholic fatty liver disease fibrosis score (NFS) than the NAFLD-only group or the non-FLD without MD group. In the MAFLD-only group and both-FLD group, the severity of the confirmed steatosis in ultrasonography was worse than that of the NAFLD-only group. The MAFLD-only group had the greatest amount (g/day) and duration of alcohol consumption and the highest rates of HBV/HCV infection and use of antiviral agents.

### Association of MAFLD and NAFLD status with incident diabetes

**Figure 1:**
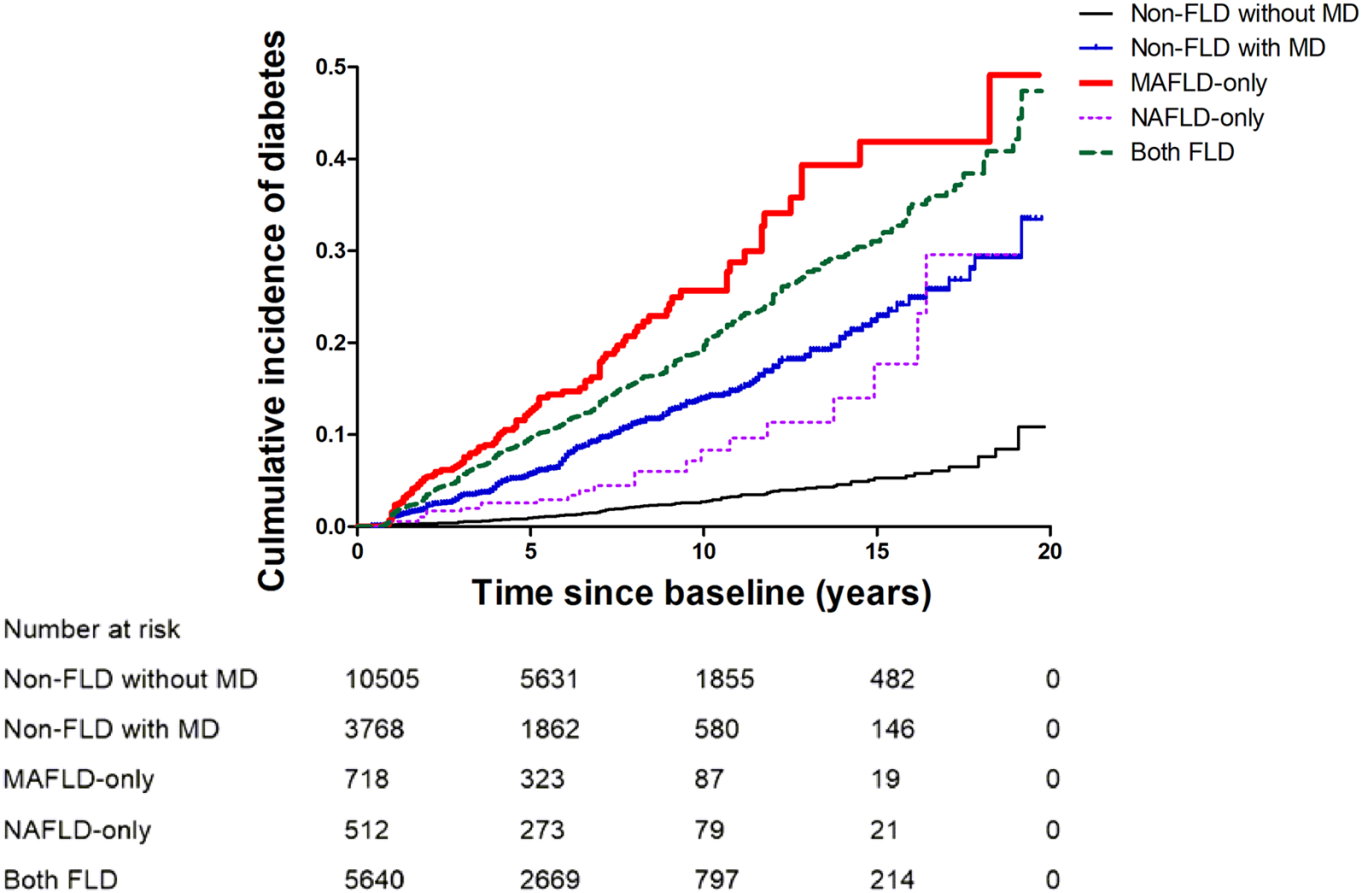
Cumulative incidence of diabetes in five patient groups (non-FLD without MD, non-FLD with MD, MAFLD-only, NAFLD-only, both FLD).

During a median 5.0 years (interquartile range, 2.4–8.2) of follow-up, 1296 incident diabetes cases occurred. The cumulative incidence of diabetes in the five groups described previously was shown in the form of Kaplan–Meier curves (Fig. 1). When the non-FLD without MD group was set as a reference, the risk of incident diabetes increased in the order of NAFLD-only (adjusted hazard ratio [aHR] 2.68, 95% confidence interval [CI], 1.58–4.53), non-FLD with MD (aHR 3.74, 95% CI 2.94–4.76), both FLD (aHR 6.17, 95% CI 4.96–7.68), and MAFLD-only (aHR 8.30, 95% CI 6.13–11.24) groups in fully adjusted model (Table 2). We also analyzed the risk of diabetes in participants with NAFLD (both FLD plus NAFLD-only group) and MAFLD (both FLD plus MAFLD-only group) compared with the non-FLD, and the aHR and 95% CI was 2.88 (2.48–3.35) in those with NAFLD and 3.13 (2.70–3.63) in those with MAFLD.

**Table 2:** Association of MAFLD and NAFLD status with incident DM.

	Cases, *n*	Events, *n*	Hazard ratio (95% CI)					
			Model 1^†^ (95%CI)	*p*	Model 2^‡^ (95% CI)	*p*	Model 3^§^ (95%CI)	*p*
Non-FLD without MD^¶^	10,505	175	Ref		Ref		Ref	
Non-FLD with MD^¶^	3768	305	5.21 (4.33–6.28)	< 0.01	4.17 (3.45–5.04)	< 0.01	3.74 (2.94–4.76)	< 0.01
Both FLD	5640	679	8.09 (6.85–9.55)	< 0.01	6.65 (5.59–7.91)	< 0.01	6.17 (4.96–7.68)	< 0.01
MAFLD-only	718	108	10.97 (8.63–13.95)	< 0.01	8.54 (6.68–10.93)	< 0.01	8.30 (6.13–11.24)	< 0.01
NAFLD-only	512	25	3.08 (2.02–4.68)	< 0.01	2.68 (1.76–4.08)	< 0.01	2.68 (1.58–4.53)	< 0.01

Abbreviations*: CI* Confidence interval; *DM* Diabetes mellitus; *CKD* Chronic kidney disease; *FLD* Fatty liver disease; *MAFLD* Metabolic dysfunction–associated fatty liver disease; *MD* Metabolic dysfunction; *NAFLD* Nonalcoholic fatty liver disease; *HDL-C* High-density lipoprotein-cholesterol; *FPG* Fasting plasma glucose; *HbA1c* Glycated hemoglobin.

^†^Model 1 was unadjusted.

^‡^Model 2 was adjusted for age and sex.

^§^Model 3 was adjusted for age, sex, smoking, physical activity, hypertension, CKD, and cardiovascular disease.

^¶^Metabolic dysfunction: the presence of two or more of the following metabolic risk abnormalities: (i) waist circumference ≥ 90 cm in men and ≥ 80 cm in women; (ii) blood pressure ≥ 130/85 mmHg or specific drug treatment; (iii) triglycerides ≥ 150 mg/dl; (iv) HDL-C < 40 mg/dl for men and < 50 mg/dl for women; (v) prediabetes (FPG 100–125 mg/dl or HbA1c 5.7–6.4%); (vi) homeostasis model assessment of insulin resistance ≥ 2.5; and (vii) high-sensitivity C-reactive protein > 2 mg/L.

Because the lower limit of the 95% CI for the aHR in the MAFLD-only group exceeded the upper limit of the 95% CI for the aHR in the non-FLD with MD and NAFLD-only groups, we sought to identify main contributors to this difference based on differences in the distribution of several risk factors for type 2 diabetes among groups at baseline (Table 1). When we further adjusted the multivariable model for alanine aminotransferase (ALT), aspartate aminotransferase (AST), low-density lipoprotein-cholesterol (LDL-C), high-density lipoprotein-cholesterol (HDL-C), TGs, FPG, and body mass index (BMI), 95% CI for the aHR in the MAFLD-only group (aHR 2.33, 95% CI 1.65–3.28) and the NAFLD-only group (aHR 2.48, 95% CI 1.47–4.21) became similar, indicating that the dominant presence of these components of MD was responsible for the higher risk of incident diabetes in the MAFLD-only group compared to the NAFLD-only group.

Next, we further analyzed the relative contributions of hepatic steatosis *per se* and of each component of MD included in the MAFLD definition to the risk of incident diabetes among those with MD with or without FLD (the MAFLD-only group and the non-FLD with MD group). In this analysis, the MAFLD-only group had a higher risk of incident diabetes than the non-FLD with MD group even in the fully adjusted model in which prediabetes was a significant independent risk factor for incident diabetes (Table 3). This indicates that hepatic steatosis *per se* contributed to the higher risk of incident diabetes in the MAFLD-only group compared to the non-FLD with MD group.

**Table 3:** Contribution of each covariate to the risk of incident diabetes in the fully adjusted Cox regression analysis model between non-FLD with MD and MAFLD-only groups.

	Model 1^†^	Model 2^‡^	Model 3^§^	Model 4^¶^	Model 5^#^
Non-FLD with MD	Ref	Ref	Ref	Ref	Ref
MAFLD-only	2.01 (1.52–2.66)	2.01 (1.52–2.66)	2.36 (1.78–3.12)	2.24 (1.68–2.99)	2.25 (1.68–3.01)
Age (in years)	1.03 (1.02–1.05)	1.03 (1.02–1.05)	1.02 (1.01–1.04)	1.02 (1.01–1.04)	1.02 (1.01–1.04)
Male sex	1.21 (0.85–1.74)	1.20 (0.83–1.73)	1.15 (0.79–1.66)	1.18 (0.81–1.70)	1.18 (0.82–1.71)
Current smoker	1.28 (0.99–1.66)	1.28 (0.98–1.66)	1.27 (0.98–1.65)	1.25 (0.96–1.63)	1.24 (0.95–1.62)
No exercise	1.23 (0.88–1.71)	1.23 (0.88–1.71)	1.37 (0.98–1.92)	1.39 (0.99–1.94)	1.38 (0.99–1.93)
Hypertension	1.14 (0.89–1.46)	1.13 (0.88–1.46)	1.28 (0.99–1.64)	1.31 (1.01–1.69)	1.30 (1.01–1.68)
CKD	1.03 (0.58–1.83)	1.03 (0.58–1.82)	1.16 (0.65–2.07)	1.14 (0.64–2.02)	1.13 (0.63–2.02)
Cardiovascular disease	0.73 (0.32–1.64)	0.73 (0.32–1.66)	1.08 (0.48–2.46)	1.07 (0.47–2.43)	1.09 (0.48–2.47)
AST	0.99 (0.98–1.01)	0.99 (0.98–1.01)	1.00 (0.98–1.01)	1.00 (0.98–1.01)	1.00 (0.98–1.01)
ALT	1.01 (1.00–1.02)	1.01 (1.00–1.02)	1.00 (0.99–1.02)	1.00 (0.99–1.01)	1.00 (0.99–1.01)
TGs ≥ 150 mg/dl		1.03 (0.80–1.32)	1.21 (0.94–1.55)	1.21 (0.94–1.56)	1.21 (0.94–1.55)
HDL-C < 40 mg/dl for men and < 50 mg/dl for women		0.94 (0.70–1.27)	1.12 (0.83–1.52)	1.14 (0.85–1.55)	1.14 (0.84–1.54)
prediabetes			6.33 (4.60–8.71)	6.50 (4.72–8.97)	6.50 (4.71–8.97)
waist circumference ≥ 90 cm in men and ≥ 80 cm in women				1.20 (0.94–1.54)	1.20 (0.93–1.54)
hs-CRP > 2 mg/L					0.48 (0.07–3.48)
HOMA-IR ≥ 2.5					1.00 (0.74–1.37)

Data are presented as adjusted hazard ratio (95% confidence interval).

Abbreviations: *CKD* Chronic kidney disease; *AST* Aspartate aminotransferase; *ALT* Alanine aminotransferase; *TGs* Triglycerides; *HDL-C* High-density lipoprotein-cholesterol; *FPG* Fasting plasma glucose; *HbA1c* Glycated hemoglobin; *hs-CRP* High-sensitivity C-reactive protein; *HOMA-IR* Homeostasis model assessment of insulin resistance; *FLD* Fatty liver disease; *MD* Metabolic dysfunction; *MAFLD* Metabolic dysfunction–associated fatty liver disease.

^†^Model 1 was adjusted for age, sex, smoking, physical activity, hypertension, CKD, cardiovascular disease, AST, and ALT.

^‡^Model 2 was adjusted for age, sex, smoking, physical activity, hypertension, CKD, cardiovascular disease, AST, ALT, TGs ≥ 150 mg/dl, and HDL-C < 40 mg/dl for men and < 50 mg/dl for women.

^§^Model 3 was adjusted for age, sex, smoking, physical activity, hypertension, CKD, cardiovascular disease, AST, ALT, TGs ≥ 150 mg/dl, HDL-C < 40 mg/dl for men and < 50 mg/dl for women, and prediabetes (FPG 100–125 mg/dl or HbA1c 5.7–6.4%).

^¶^Model 4 was adjusted for age, sex, smoking, physical activity, hypertension, CKD, cardiovascular disease, AST, ALT, TGs ≥ 150 mg/dl, HDL-C < 40 mg/dl for men and < 50 mg/dl for women, prediabetes (FPG 100–125 mg/dl or HbA1c 5.7–6.4%), and waist circumference ≥ 90 cm in men and ≥ 80 cm in women.

^**#**^Model 5 was adjusted for age, sex, smoking, physical activity, hypertension, CKD, cardiovascular disease, AST, ALT, TGs ≥ 150 mg/dl, HDL-C < 40 mg/dl for men and < 50 mg/dl for women, prediabetes (FPG 100–125 mg/dl or HbA1c 5.7–6.4%), waist circumference ≥ 90 cm in men and ≥ 80 cm in women, HOMA-IR ≥ 2.5, and hs-CRP > 2 mg/L.

### Association between MAFLD and NAFLD status and incident diabetes according to BMI category, presence of MD, alcohol intake and HBV/HCV infection

Participants with MAFLD had a higher risk of developing diabetes than those without MAFLD regardless of overweight/obesity status (BMI < 23 kg/m^2^ and ≥ 23 kg/m^2^). These results were also consistent when the participants with NAFLD were stratified by overweight/obesity with those without NAFLD (Supplementary Table 1). We divided the participants with MAFLD into three subgroups according to the presence of excessive alcohol consumption and HBV/HCV infection, and all three subgroups had a significantly increased risk of incident diabetes (MAFLD with MD only, aHR 7.63, 95% CI 6.03–9.66; MAFLD with excessive alcohol consumption group, aHR 9.39, 95% CI 6.47–13.62; MAFLD with HBV/HCV infection groups, HR 6.06, 95% CI 3.76–9.77) (Supplementary Table 2).

### Synergistic effect among the presence of excessive alcohol consumption and/or HBV/HCV infection, hepatic steatosis, and MD on the risk of incident diabetes

We further explored whether there was a synergistic effect among the presence of excessive alcohol consumption, hepatic steatosis, and MD on the risk of incident diabetes. Excessive alcohol intake itself did not increase the risk of incident diabetes when hepatic steatosis and MD were absent (aHR 0.65, 95% CI 0.21–2.06). However, the presence of both excessive alcohol intake and MD (aHR 5.21, 95% CI 3.18–8.55) and of both hepatic steatosis and MD (aHR 7.88, 95% CI 6.22–9.98) significantly increased the risk of incident diabetes. The presence of all three components (excessive alcohol consumption, hepatic steatosis, and MD) synergistically increased the risk of incident diabetes (aHR 11.92, 95% CI 8.39–16.95, Model 3, Table 4). The results were consistent when we adjusted the amount and duration of alcohol consumption in each multivariable analysis model (Model 4, Table 4).

**Table 4:** Association of excessive alcohol consumption, fatty liver disease, and metabolic dysfunction with incident DM.

	Cases, *n*	Events, *n*	Hazard ratio (95% CI)							
			Model 1^† ^(95%CI)	*p*	Model 2^‡ ^(95%CI)	*p*	Model 3^§ ^(95%CI)	*p*	Model 4^¶^ (95%CI)	*p*
Alcohol(−)MD(−)FLD(−)	10,125	169	Ref		Ref		Ref		Ref	
Alcohol( +)MD(−)FLD(−)	380	6	1.12 (0.50–2.53)	0.78	0.98 (0.43–2.21)	0.95	0.65 (0.21–2.06)	0.47	0.90 (0.26–3.08)	0.87
Alcohol( +)MD( +)FLD(−)	277	24	6.39 (4.16–9.79)	< 0.01	4.87 (3.15–7.52)	< 0.01	5.21 (3.18–8.55)	< 0.01	5.91 (2.91–12.00)	< 0.01
Alcohol(−)MD( +)FLD( +)	4236	653	10.52 (8.89–12.47)	< 0.01	8.36 (6.98–10.03)	< 0.01	7.88 (6.22–9.98)	< 0.01	7.94 (6.00–10.51)	< 0.01
Alcohol( +)MD( +)FLD( +)	347	68	15.50 (11.69–20.55)	< 0.01	11.78 (8.80–15.78)	< 0.01	11.92 (8.39–16.95)	< 0.01	13.06 (7.13–23.91)	< 0.01

Abbreviations: *CI* Confidence interval; *CKD* Chronic kidney disease; *FLD* Fatty liver disease; *DM*, Diabetes mellitus; *MD* Metabolic dysfunction.

^†^Model 1 was unadjusted.

^‡^Model 2 was adjusted for age and sex.

^§^Model 3 was adjusted for age, sex, smoking, physical activity, hypertension, CKD, and cardiovascular disease.

^¶^Model 4 was adjusted for age, sex, smoking, physical activity, hypertension, CKD, cardiovascular disease, and amount and duration of alcohol.

These results were similar when we explored the effects of HBV/HCV infection, hepatic steatosis, and MD on the risk of incident diabetes (Table 5). The results were consistent when we adjusted for use of antiviral agents in each multivariable analysis model (Model 4, Table 5).

**Table 5:** Association of HBV/HCV infection, fatty liver disease, and metabolic dysfunction with incident DM.

	Cases, *n*	Events, *n*	Hazard ratio (95% CI)							
			Model 1^†^ (95%CI)	*p*	Model 2^‡^ (95%CI)	*p*	Model 3^§^ (95%CI)	*p*	Model 4^¶^ (95%CI)	*p*
HBV/HCV(−)MD(−)FLD(−)	9935	163	Ref		Ref		Ref		Ref	
HBV/HCV( +)MD(−)FLD(−)	570	12	1.30 (0.72–2.34)	0.38	1.21 (0.67–2.18)	0.52	1.05 (0.49–2.27)	0.89	1.05 (0.48–2.29)	0.90
HBV/HCV( +)MD( +)FLD(−)	232	26	7.04 (4.65–10.65)	< 0.01	5.65 (3.72–8.57)	< 0.01	6.11 (3.59–10.39)	< 0.01	6.09 (3.45–10.76)	< 0.01
HBV/HCV(−)MD( +)FLD( +)	4381	686	10.91 (9.19–12.94)	< 0.01	8.64 (7.20–10.36)	< 0.01	8.42 (6.64–10.67)	< 0.01	8.42 (6.64–10.67)	< 0.01
HBV/HCV( +)MD( +)FLD( +)	202	35	12.89 (8.94–18.58)	< 0.01	10.08 (6.95–14.60)	< 0.01	8.61 (5.28–14.03)	< 0.01	8.59 (5.07–14.56)	< 0.01

Abbreviations: *CI* Confidence interval; *CKD* Chronic kidney disease; *FLD* Fatty liver disease; *DM* Diabetes mellitus; *MD* Metabolic dysfunction; *HBV* Hepatitis B virus; *HCV* Hepatitis C virus.

^†^Model 1 was unadjusted.

^‡^Model 2 was adjusted for age and sex.

^§^Model 3 was adjusted for age, sex, smoking, physical activity, hypertension, CKD, and cardiovascular disease.

^¶^Model 4 was adjusted for age, sex, smoking, physical activity, hypertension, CKD, cardiovascular disease, and use of antiviral agents.

## Discussion

In this retrospective longitudinal cohort study of 21,178 adults, the risk of incident diabetes increased in the order of NAFLD-only, non-FLD with MD, both FLD, and MAFLD-only, using the non-FLD without MD group as a reference. The lower limit of the 95% CI for HR of diabetes incidence rate in the both FLD or MAFLD-only groups did not overlap the upper limit of the 95% CI for HR in the NAFLD-only or non-FLD with MD groups. The MAFLD-only group, which included subjects with MD or excessive alcohol consumption, had a higher risk of incident diabetes than the NAFLD-only group. In the subgroup analysis of the MAFLD group according to the presence of MD only, excessive alcohol consumption, and HBV/HCV infection, all three subgroups had a significantly increased risk of incident diabetes. A synergistic effect of the presence of excessive alcohol intake and/or HBV/HCV infection, hepatic steatosis, and MD on the risk of incident diabetes was also identified.

After the MAFLD definition was proposed, researchers conducted studies to validate the performance of the MAFLD and NAFLD criteria in terms of the prediction of the risk of extra-hepatic outcomes. Although a few previous studies have shown a difference of diabetes prevalence in individuals with MAFLD and NAFLD, most of the studies were cross-sectional and compared diabetes prevalence rather than incidence^17,30–32^. One longitudinal cohort study reported that both NAFLD and MAFLD increased the incidence of diabetes similarly compared with non-FLD group^17^. In our study, however, we focused on the comparison of MAFLD-only or NAFLD-only groups, in which the change in FLD definition would be clinically relevant, and revealed that the MAFLD-only group had a higher risk for incident diabetes than the NAFLD-only group. This difference in the study design, along with an improved power in the analyses in the MAFLD-only population compared with previous studies^17,30–32^, would explain the clearer difference between the two FLD definitions in the current study.

Moreover, our study revealed that the MAFLD-only group had a higher risk of developing diabetes than the non-FLD with MD group, members of which have the same MD background as in the MAFLD-only group but not in those with FLD. In addition, we investigated whether the higher risk in the MAFLD-only group was due to FLD or the MD component. In this study, presence of FLD *per se* remained a significant contributor to the increased risk of diabetes even after adjustment for all components of MD in the subgroup analysis restricted in those with MD (Table 3). This supports the hypothesis that FLD *per se* has a role in diabetes development beyond the background MD in this subgroup. These results add on the findings of the interaction between alcohol consumption, presence of FLD, and risk of diabetes observed in previous studies conducted in the general population with or without MD^23^. These results are also in line with results from a recent longitudinal cohort study reporting a higher HR for incident diabetes in the MAFLD group compared with simple FLD and non-FLD with MD groups^29^. However, the previous study did not specifically explore the risk of diabetes in the MAFLD-only group^29^.

The MAFLD-only group had a higher risk of diabetes than the non-FLD with MD group and the NAFLD-only group, without overlapping of 95% CIs. When we confined the analysis to MAFLD with excessive alcohol intake or MAFLD with HBV/HCV infection, which would have been excluded from the NAFLD definition, the increased risk of incident diabetes remained significant. Because previous studies have suggested that excessive alcohol intake^23–25^ and HBV/HCV infection^26–28^ are risk factors for diabetes, we questioned whether there was a synergistic effect of the presence of excessive alcohol consumption and/or HBV/HCV infection, hepatic steatosis, and MD on the risk of incident diabetes. Indeed, the presence of excessive alcohol intake and/or HBV/HCV infection significantly increased the risk of incident diabetes only when MD and/or FLD were present. The HRs showed a synergistic effect of MD and hepatic steatosis in the presence of excessive alcohol intake and/or HBV/HCV infection.

The mechanism of increased risk of diabetes in the both-FLD group may be similar to that of NAFLD, which is associated with increased risk of diabetes^2–4,7,8,14,33^. However, less attention has been paid to the mechanism of increased risk of diabetes in MAFLD-only patients, which included individuals with excessive alcohol consumption or HBV/HCV infection. A possible explanation is that alcohol intake^34^ or HBV/HCV infection^35,36^ causes liver injury and leads to advanced hepatic fibrosis, which is associated with impaired glucose tolerance. Indeed, the MAFLD-only group in this study showed a higher NFS and increased severity of steatosis in ultrasound compared with the NAFLD-only group.

The strength of this study was that all study subjects underwent standardized abdominal ultrasonography in a large scale. Therefore, we evaluated the incidence of diabetes for the patients who had ultrasound-diagnosed fatty liver. The relatively large sample size allowed us to examine the incidence of diabetes in stratified subgroup analysis to evaluate the synergistic effects of alcohol or HBC/HCV infection and MD and FLD.

This study also had several limitations. First, this is an observational study, in which clarifying the causal relationship is difficult to establish. Second, subjects with diabetes were excluded in the study because diabetes, which was included in the MAFLD diagnostic criteria, was the outcome of study. Third, it may be difficult to generalize the results of the study to the general population, because the majority of the study subjects were Korean healthy male workers who had health check-up examinations at work annually or biannually. Finally, hepatic steatosis was diagnosed by abdominal ultrasonography, not liver biopsy, which is the gold standard test. However, ultrasonography has been recognized as a reliable non-invasive method for diagnosing fatty liver^37^.

In conclusion, the MAFLD-only group showed a greater increase in incidence of diabetes than the NAFLD-only and non-FLD with MD groups. The presence of excessive alcohol consumption and/or HBV/HCV infection, which excluded from NAFLD diagnostic criteria, along with hepatic steatosis and MD may synergistically increase the risk of incident diabetes, indicating that hepatic steatosis in these categories should also be considered as an indicator of increased risk of incident diabetes.

## Methods

### Study population

**Figure 2:**
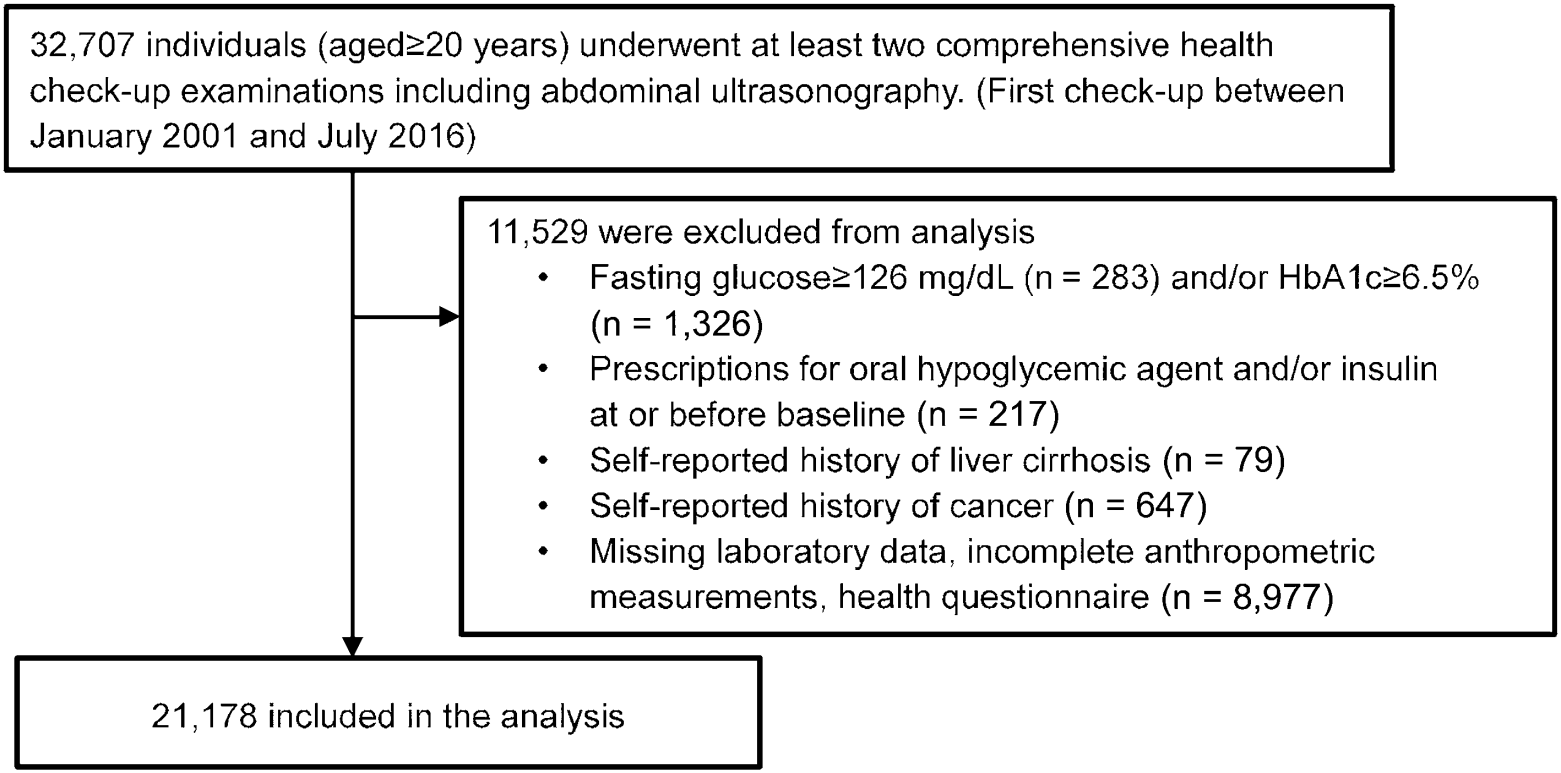
Flow diagram of the study population.

This study included individuals (aged ≥ 20 years) who underwent at least two comprehensive health examinations including abdominal ultrasonography at the Health Promotion Center at Samsung Medical Center (SMC, Seoul, Republic of Korea). Patients with FPG ≥ 126 mg/dl or HbA1c ≥ 6.5% or taking anti-diabetic medications at baseline were excluded. Patients with any history of cancer or liver cirrhosis were also excluded. Individuals with missing data such as laboratory or anthropometric measurements or health questionnaires were excluded (Fig. 2).

A total of 21,178 adults were included in the study, of which 14,273 had no hepatic steatosis on abdominal ultrasonography. Those without FLD were categorized into two groups according to the presence of MD: 1) individuals without MD (non-FLD without MD) (n = 10,505) and 2) individuals with MD (non-FLD with MD) (n = 3,768). Participants with FLD on abdominal ultrasonography were categorized into three groups: 1) individuals who met both MAFLD and NAFLD criteria (both FLD) (n = 5,640), 2) individuals who were not previously classified as NAFLD but met MAFLD criteria (MAFLD-only) (n = 718), and 3) individuals who were previously classified as NAFLD but did not meet MAFLD criteria (NAFLD-only) (n = 512). In additional analyses, MAFLD was further categorized into two groups according to body mass index (BMI) (< 23 kg/m^2^ and ≥ 23 kg/m^2^) or three groups according to presence of MD only, excessive alcohol intake, and HBV/HCV infection (regardless of MD in the latter two groups). To assess the interactions between excessive alcohol intake, HBV/HCV infection, MD, and FLD, individuals with excessive alcohol consumption or HBV/HCV infection were divided into groups according to MD and FLD.

The study protocol was approved by the Institutional Review Board (IRB) of SMC (no. 2021–05-025). The informed consent requirement was waived by the IRB because the study information was de-identified. The protocol for the study was in accordance with the guidelines of the Declaration of Helsinki.

### Clinical variables, measurements, and definitions

The health checkup examination collected information on medical history, smoking status, alcohol status, exercise status, medication, anthropometric data and laboratory data. A self-administered questionnaire was used to collect data about medical history, prescribed medications, alcohol consumption, smoking and exercise history. The study population were asked about average daily intake of alcohol, duration of lifetime alcohol intake. Excessive alcohol consumption was defined as more than 30 g daily for men and more than 20 g daily for women. Smoking status was categorized as never, former smoker, or current smoker. Exercise status was categorized as none, 1–2 days per week, 3–4 days per week or ≥ 5 days per week. BMI was calculated as weight divided by height squared (kg/m^2^). Diabetes was defined as FPG ≥ 126 mg/dl or HbA1c ≥ 6.5% or taking medication for diabetes. Hypertension was defined as blood pressure ≥ 130/85 mmHg or taking medication for hypertension. CKD was defined as eGFR less than 60 mL/min/1.73 m^2^ calculated by the Chronic Kidney Disease Epidemiology Collaboration (CKD-EPI) formula or uACR greater than or equal to 30 mg/g. Venous blood samples were collected after an overnight fast of at least 12 h. Insulin level was measured using an immunoradiometric assay (DIAsource Co., Louvain-la-Neuve, Belgium). HOMA-IR was calculated as FPG (mg/dl) x fasting plasma insulin (uIU/mL)/405^38^. HBV infection was defined as positive hepatitis B virus surface antigen (HBsAg) or a history of antiviral treatment. HCV infection was defined as positive hepatitis C antibody (anti-HCV Ab) or a history of antiviral treatment. An ultrasound-based four-grade scale was used to evaluate liver steatosis. Fatty liver was categorized as normal, mild, moderate or severe^37,39^. Abdominal ultrasonography was performed by experienced ultrasonographists.

### Definition of MAFLD and NAFLD

MAFLD was diagnosed based on ultrasound evidence of fatty liver with one of the following 3 criteria: overweight/obesity (defined as BMI ≥ 23.0 kg/m^2^ for Asians), diabetes or MD^15^. Since we excluded patients with pre-existing diabetes, MAFLD was defined as satisfying either category of body weight or MD. MD was defined as the presence of at least 2 of the following metabolic abnormalities: 1) waist circumference (WC) ≥ 90 cm in men and ≥ 80 cm in women; 2) blood pressure ≥ 130/85 mmHg or specific drug treatment; 3) plasma TGs level ≥ 150 mg/dl or specific drug treatment; 4) plasma HDL-C level < 40 mg/dl for men and < 50 mg/dl for women or specific drug treatment; 5) prediabetes (FPG level 100–125 mg/dl or HbA1c 5.7%–6.4%; 6) HOMA-IR ≥ 2.5; or 7) plasma hs-CRP level > 2 mg/L.

NAFLD was defined as the presence of hepatic steatosis in the absence of excessive alcohol consumption and other causes of liver diseases^40–42^.

### Study outcome

The endpoint of this study was development of new-onset diabetes. New-onset diabetes was defined by one of three conditions: 1) FPG ≥ 126 mg/dl, 2) HbA1c ≥ 6.5%, or 3) taking anti-diabetic medication^43,44^.

### Statistical analysis

Statistical analyses were performed using SPSS, version 27.0 (SPSS Inc.). The baseline characteristics of the study population were compared in the five groups (non-FLD without MD, non-FLD with MD, MAFLD-only, NAFLD-only, or both FLD). Continuous variables with normal distributions are shown as the mean ± standard deviation and categorical data are presented as frequencies and percentages.

Multivariate Cox regression analysis was performed to calculate the HRs and 95% CIs for the outcome incidence rates: unadjusted in model 1, adjusted for age and sex in model 2, and adjusted for age, sex, smoking history, regular exercise and presence of HTN, CKD, cardiovascular disease in model 3. Kaplan–Meier curves were used to compare the incidence of diabetes in the five groups (non-FLD without MD, non-FLD with MD, MAFLD-only, NAFLD-only, both FLD).

To evaluate synergistic effects between excessive alcohol intake or HBV/HCV infection and MD and FLD, two separate subgroup analyses were also conducted according to 1) excessive alcohol consumption and 2) HBV/HCV infection.

## Supplementary Information

Supplementary Information.

## Data Availability

The datasets generated and/or analyzed during this study are available from the corresponding authors on reasonable request.

## Abbreviations

NAFLD: Nonalcoholic fatty liver disease
MAFLD: Metabolic dysfunction-associated fatty liver disease
MD: Metabolic dysfunction
HBV: Hepatitis B virus
HCV: Hepatitis C virus
FPG: Fasting plasma glucose
HbA1c: Glycosylated hemoglobin
FLD: Fatty liver disease
BMI: Body mass index
CKD: Chronic kidney disease
HOMA-IR: Homeostasis model assessment of insulin resistance
IRB: Institutional review board
TG: Triglyceride
LDL-C: Low-density lipoprotein-cholesterol
HDL-C: High-density lipoprotein-cholesterol
HR: Hazard ratio
CI: Confidence interval
WC: Waist circumference
NFS: Nonalcoholic fatty liver disease fibrosis score
eGFR: Estimated glomerular filtration rate
uACR: Urinary albumin-to-creatinine ratio
hs-CRP: High-sensitivity C-reactive protein
ALT: Alanine aminotransferase
AST: Aspartate aminotransferase

## References

[1] Anstee QM, Targher G, Day CP (2013). Progression of NAFLD to diabetes mellitus, cardiovascular disease or cirrhosis. Nat Rev Gastroenterol Hepatol.

[2] Yki-Järvinen H (2014). Non-alcoholic fatty liver disease as a cause and a consequence of metabolic syndrome. Lancet Diabetes Endocrinol.

[3] Cusi K (2017). Non-alcoholic fatty liver disease (NAFLD) prevalence and its metabolic associations in patients with type 1 diabetes and type 2 diabetes. Diabetes Obes Metab.

[4] Mantovani A (2021). Non-alcoholic fatty liver disease and risk of incident diabetes mellitus: an updated meta-analysis of 501 022 adult individuals. Gut.

[5] Xia MF, Bian H, Gao X (2019). NAFLD and diabetes: two sides of the same coin? Rationale for gene-based personalized NAFLD treatment. Front Pharmacol.

[6] Lee YH (2019). Nonalcoholic fatty liver disease in diabetes. Part I: epidemiology and diagnosis. Diabetes Metab J.

[7] Lonardo A, Nascimbeni F, Mantovani A, Targher G (2018). Hypertension, diabetes, atherosclerosis and NASH: cause or consequence?. J Hepatol.

[8] Lonardo A (2019). A round trip from nonalcoholic fatty liver disease to diabetes: molecular targets to the rescue?. Acta Diabetol.

[9] Aragón JJ, Martínez-Costa OH (2014). Ectopic fat in insulin resistance, dyslipidemia, and cardiometabolic disease. N Engl J Med.

[10] Byrne CD (2013). Ectopic fat, insulin resistance and non-alcoholic fatty liver disease. Proc Nutr Soc.

[11] Fontana L, Eagon JC, Trujillo ME, Scherer PE, Klein S (2007). Visceral fat adipokine secretion is associated with systemic inflammation in obese humans. Diabetes.

[12] Misu H (2010). A liver-derived secretory protein, selenoprotein P, causes insulin resistance. Cell Metab.

[13] Jung CH (2013). Associations of serum fetuin-A levels with insulin resistance and vascular complications in patients with type 2 diabetes. Diab Vasc Dis Res.

[14] Targher G, Corey KE, Byrne CD, Roden M (2021). The complex link between NAFLD and type 2 diabetes mellitus - mechanisms and treatments. Nat Rev Gastroenterol Hepatol.

[15] Eslam M (2020). A new definition for metabolic dysfunction-associated fatty liver disease: an international expert consensus statement. J Hepatol.

[16] Younossi ZM (2019). Non-alcoholic fatty liver disease - a global public health perspective. J Hepatol.

[17] Liang Y (2022). Association of MAFLD with diabetes, chronic kidney disease, and cardiovascular disease: a 4.6-year cohort study in China. J Clin Endocrinol Metab.

[18] Valenzuela-Vallejo L, Mantzoros CS (2022). Time to transition from a negative nomenclature describing what NAFLD is not, to a novel, pathophysiology-based, umbrella classification of fatty liver disease (FLD). Metabolism.

[19] Joo EJ, Chang Y, Yeom JS, Ryu S (2017). Hepatitis B virus infection and decreased risk of nonalcoholic fatty liver disease: a cohort study. Hepatology.

[20] Mak LY, Yuen MF, Seto WK (2020). Letter regarding "A new definition for metabolic dysfunction-associated fatty liver disease: an international expert consensus statement". J Hepatol.

[21] Lonardo A (2016). Fatty liver is associated with an increased risk of diabetes and cardiovascular disease - evidence from three different disease models: NAFLD, HCV and HIV. World J Gastroenterol.

[22] Lin S (2021). Concurrence of HBV infection and non-alcoholic fatty liver disease is associated with higher prevalence of chronic kidney disease. Clin Res Hepatol Gastroenterol.

[23] Okamura T (2020). Effect of alcohol consumption and the presence of fatty liver on the risk for incident type 2 diabetes: a population-based longitudinal study. BMJ Open Diabetes Re Care.

[24] Kao WH, Puddey IB, Boland LL, Watson RL, Brancati FL (2001). Alcohol consumption and the risk of type 2 diabetes mellitus: atherosclerosis risk in communities study. Am J Epidemiol.

[25] Lee DY (2017). Association between alcohol consumption pattern and the incidence risk of type 2 diabetes in Korean men: a 12-years follow-up study. Sci Rep.

[26] Cai C (2015). Association between hepatitis B virus infection and diabetes mellitus: a meta-analysis. Exp Ther Med.

[27] Khalili M (2015). Diabetes and prediabetes in patients with hepatitis B residing in North America. Hepatology.

[28] White DL, Ratziu V, El-Serag HB (2008). Hepatitis C infection and risk of diabetes: a systematic review and meta-analysis. J Hepatol.

[29] Miyake T (2022). Fatty liver with metabolic disorder, such as metabolic dysfunction-associated fatty liver disease, indicates high risk for developing diabetes mellitus. J Diabetes Investig.

[30] Lim GEH (2021). An observational data meta-analysis on the differences in prevalence and risk factors between MAFLD vs NAFLD. Clin Gastroenterol Hepatol..

[31] Lin S (2020). Comparison of MAFLD and NAFLD diagnostic criteria in real world. Liver Int.

[32] Yu C (2022). Comparing the diagnostic criteria of MAFLD and NAFLD in the Chinese population: a population-based prospective cohort study. J Clin Transl Hepatol.

[33] Shibata M, Kihara Y, Taguchi M, Tashiro M, Otsuki M (2007). Nonalcoholic fatty liver disease is a risk factor for type 2 diabetes in middle-aged Japanese men. Diabetes Care.

[34] Mitsuhashi K (2017). Impact of fatty liver disease and metabolic syndrome on incident type 2 diabetes; a population based cohort study. Endocr J.

[35] Allison ME, Wreghitt T, Palmer CR, Alexander GJ (1994). Evidence for a link between hepatitis C virus infection and diabetes mellitus in a cirrhotic population. J Hepatol.

[36] Lonardo A, Adinolfi LE, Petta S, Craxì A, Loria P (2009). Hepatitis C and diabetes: the inevitable coincidence?. Expert Rev Anti Infect Ther.

[37] Hernaez R (2011). Diagnostic accuracy and reliability of ultrasonography for the detection of fatty liver: a meta-analysis. Hepatology.

[38] Matthews DR (1985). Homeostasis model assessment: insulin resistance and beta-cell function from fasting plasma glucose and insulin concentrations in man. Diabetologia.

[39] Dasarathy S (2009). Validity of real time ultrasound in the diagnosis of hepatic steatosis: a prospective study. J Hepatol.

[40] Watanabe S (2015). Evidence-based clinical practice guidelines for nonalcoholic fatty liver disease/nonalcoholic steatohepatitis. Hepatol Res.

[41] Chalasani N (2012). The diagnosis and management of non-alcoholic fatty liver disease: Practice guideline by the American association for the study of liver diseases, American college of gastroenterology, and the American gastroenterological association. Am J Gastroenterol.

[42] Sinn DH (2022). Nonalcoholic fatty liver disease without metabolic-associated fatty liver disease and the risk of metabolic syndrome. Clin Gastroenterol Hepatol..

[43] American Diabetes Association (2021). 2. classification and diagnosis of diabetes: standards of medical care in diabetes-2021. Diabetes Care.

[44] Yoo JH (2021). Mean and visit-to-visit variability of glycated hemoglobin, and the risk of non-alcoholic fatty liver disease. J Diabetes Investig.

