# Supplementary material for: Increased risk of incident diabetes in patients with MAFLD not meeting the criteria for NAFLD
Source: Sci Rep. 2023 Jul 1;13:10677. doi: 10.1038/s41598-023-37858-8 (PMC10314928; doi:10.1038/s41598-023-37858-8)
Supplement: Supplementary file 1 — Supplementary Information. [file 41598_2023_37858_MOESM1_ESM.docx]

**Table S1.**  Association between BMI and incident DM in subjects with MAFLD or NAFLD.

|  | **Cases,**  ***n*** | **Events,**  ***n*** | **Hazard ratio (95% CI)** | | | | | |
| --- | --- | --- | --- | --- | --- | --- | --- | --- |
|  |  |  | **Model 1^†^ (95%CI)** | ***p*** | **Model 2^‡^ (95%CI)** | ***p*** | **Model 3^§^ (95%CI)** | ***p*** |
| No MAFLD | 14,820 | 509 | Ref |  | Ref |  | Ref |  |
| MAFLD with BMI <23kg/m² | 383 | 56 | 4.59 (3.48–6.04) | <0.01 | 3.51 (2.66–4.64) | <0.01 | 2.91 (2.00–4.23) | <0.01 |
| MAFLD with BMI≥23kg/m² | 5,975 | 731 | 3.95 (3.53–4.43) | <0.01 | 3.30 (2.93–3.71) | <0.01 | 3.10 (2.67–3.59) | <0.01 |
| No NAFLD | 15,026 | 592 | Ref |  | Ref |  | Ref |  |
| NAFLD with BMI <23kg/m² | 855 | 78 | 2.40 (1.89–3.03) | <0.01 | 2.00 (1.58–2.54) | <0.01 | 1.89 (1.39–2.56) | <0.01 |
| NAFLD with BMI ≥23kg/m² | 5,297 | 626 | 3.28 (2.93–3.67) | <0.01 | 2.72 (2.42–3.05) | <0.01 | 2.50 (2.17–2.89) | <0.01 |

^†^Model 1 was unadjusted.

^‡^Model 2 was adjusted for age and sex.

^§^Model 3 was adjusted for age, sex, smoking, physical activity, hypertension, CKD, and cardiovascular disease.

Abbreviations: BMI, body mass index; CI, confidence interval; CKD, chronic kidney disease; DM, diabetes mellitus; MD, metabolic dysfunction; MAFLD, metabolic dysfunction–associated fatty liver disease; NAFLD, nonalcoholic fatty liver disease.

**Table S2**. Associations of excessive alcohol intake and HBV/HCV infection with incident DM in subjects with MAFLD.

|  | **Cases,**  ***n*** | **Events,**  ***n*** | **Hazard ratio (95% CI)** | | | | | |
| --- | --- | --- | --- | --- | --- | --- | --- | --- |
|  |  |  | **Model 1^†^ (95%CI)** | ***p*** | **Model 2^‡^ (95% CI)** | ***p*** | **Model 3^§^ (95%CI)** | ***p*** |
| Non-FLD without MD | 10,505 | 175 | Ref |  | Ref |  | Ref |  |
| Non-FLD with MD | 3,768 | 305 | 5.23 (4.34–6.30) | <0.01 | 4.04 (3.34–4.90) | <0.01 | 4.40 (3.40–5.70) | <0.01 |
| MAFLD | 6,358 | 787 | 8.38 (7.11–9.87) | <0.01 | 6.82 (5.72–8.12) | <0.01 | 6.27 (5.01–7.84) | <0.01 |
| With MD only^¶^ | 4,047 | 620 | 10.31 (8.72–12.18) | <0.01 | 8.13 (6.80–9.72) | <0.01 | 7.63 (6.03–9.66) | <0.01 |
| With excessive alcohol intake^#^ | 429 | 71 | 12.58 (9.54–16.60) | <0.01 | 8.43 (6.30–11.28) | <0.01 | 9.39 (6.47–13.62) | <0.01 |
| With HBV/HCV infection | 305 | 39 | 9.17 (6.47–12.98) | <0.01 | 6.72 (4.70–9.60) | <0.01 | 6.06 (3.76–9.77) | <0.01 |

^†^Model 1 was unadjusted.

^‡^Model 2 was adjusted for age and sex.

^§^Model 3 was adjusted for age, sex, smoking, physical activity, hypertension, CKD, and cardiovascular disease.

^¶^Subjects with MAFLD who did not drink excessively and did not have HBV/HCV infection.

^#^Excess consumption of alcohol was defined as more than 30 g daily of alcohol consumption in men and more than 20 g in women.

Abbreviations: CI, confidence interval; CKD, chronic kidney disease; DM, diabetes mellitus; MD, metabolic dysfunction; MAFLD, metabolic dysfunction–associated fatty liver disease; HBV, hepatitis B virus; HCV, hepatitis C virus.
